# Views by health professionals on the responsiveness of commune health stations regarding non-communicable diseases in urban Hanoi, Vietnam: a qualitative study

**DOI:** 10.1186/s12913-018-3217-4

**Published:** 2018-05-31

**Authors:** Vu Duy Kien, Hoang Van Minh, Kim Bao Giang, Nawi Ng, Viet Nguyen, Le Thanh Tuan, Malin Eriksson

**Affiliations:** 1 Oncare Medical Technology Company Limited , Hanoi, Vietnam; 2 Center for Population Health Sciences, Hanoi University of Public Health, Hanoi, Vietnam; 3 Unit of Epidemiology and Global Health, Department of Public Health and Clinical Medicine, Umeå University, Umeå, Sweden; 4 Institute for Preventive Medicine and Public Health, Hanoi Medical University, Hanoi, Vietnam; 5 Harvard Medical School, Boston, MA USA; 6 Department of Training and Management, Thanh Hoa Medical College, Thanh Hoa, Vietnam

**Keywords:** Non-communicable disease, Responsiveness, Commune health station, Urban, Vietnam

## Abstract

**Background:**

Primary health care plays an important role in addressing the burden of non-communicable diseases (NCDs) in low- and middle-income countries. In light of the rapid urbanization of Vietnam, this study aims to explore health professionals’ views about the responsiveness of primary health care services at commune health stations, particularly regarding the increase of NCDs in urban settings.

**Methods:**

This qualitative study was conducted in Hanoi from July to August 2015. We implemented 19 in-depth interviews with health staff at four purposely selected commune health stations and conducted a brief inventory of existing NCD activities at these commune health stations. We also interviewed NCD managers at national, provincial, and district levels. The interview guides reflected six components of the WHO health system framework, including service delivery, health workforce, health information systems, access to essential medicines, financing, and leadership/governance. A thematic analysis approach was applied to analyze the interview data in this study.

**Results:**

Six themes, related to the six building blocks of the WHO health systems framework, were identified. These themes explored the responsiveness of commune health stations to NCDs in urban Hanoi. Health staff at commune health stations were not aware of the national strategy for NCDs. Health workers noted the lack of NCD informational materials for management and planning. The limited workforce at health commune stations would benefit from more health workers in general and those with NCD-specific training and skills. In addition, the budget for NCDs at commune health stations remains very limited, with large differences in the implementation of national targeted NCD programs. Some commune health stations had no NCD services available, while others had some programming. A lack of NCD treatment drugs was also noted, with a negative impact on the provision of NCD-related services at commune health stations. These themes were also reflected in the inventory of existing NCD related activities.

**Conclusions:**

Health professionals view the responsiveness of commune health stations to NCDs in urban Hanoi, Vietnam as weak. Appropriate policies should be implemented to improve the primary health care services on NCDs at commune health stations in urban Hanoi, Vietnam.

**Electronic supplementary material:**

The online version of this article (10.1186/s12913-018-3217-4) contains supplementary material, which is available to authorized users.

## Background

Non-communicable diseases (NCDs), namely cardiovascular diseases, diabetes, cancer and chronic respiratory diseases, caused 38 million deaths globally in 2012. Most deaths (28 million) occurred in low- and middle- income countries (LMICs) [1]. The annual loss attributable to NCDs amounted to approximately 4% of GDP for LMICs [2]. In addition, rapid urbanization challenged population health in developing countries [3]. This has been accompanied by an increase in urban poverty and the development of slums [4, 5]. Populations afflicted with poverty and particularly those living in slums are the most vulnerable urban groups, bearing a double burden of both communicable and non-communicable diseases [6]. Primary health care services are vital to addressing NCDs, especially in LMICs [7]. The World Health Organization also highlighted the role of primary health care systems to execute NCD interventions [8, 9].

Vietnam has achieved significant results in improving population health. However, like other LMICs, Vietnam has suffered from a double burden of disease, in that the burden of communicable disease remains, while the burden of non-communicable diseases (NCD) is increasing [10]. The national hospital records showed that the proportion of communicable diseases decreased from 55.5 to 25.3% between 1970 and 2013 while the proportion of NCDs increased from 42.7 to 63.5% during the same period [11]. To address this, the Vietnamese government has approved and implemented a specific national target program for NCDs in 2002. Currently, this program received renewed approval for 2015–2025 and includes CVDs, diabetes, cancer, respiratory lung diseases (including chronic obstructive pulmonary diseases/COPDs and asthma) and other NCDs [12]. To implement the national strategy, several vertical programs were established that include programs on 1) hypertension prevention and control, 2) cancer prevention and control, 3) diabetes prevention and control, 4) COPDs and asthma prevention and control, and 5) protection of mental health in the community and among children. Although these national targeted programs were established quite early, budget constraints limited the scale of the initial pilot activities. The programs on hypertension, diabetes and mental health were implemented at the commune level through a selection of 4–5 commune health stations per district. The programs on cancer, COPDs and asthma were implemented at provincial and district levels. All the above-mentioned programs focused mainly on improving communication and screening services [12].

In Vietnam, a commune health station is the lowest level in the health system, and the closest to the community in terms of providing primary health care services [11]. In addition, the commune health station is involved in many national target programs, such as immunization, nutrition, tuberculosis, family planning, HIV, environmental and food safety. The number of health staff in a commune health station is based on the commune’s population, with a range of 5 to 10 health staff per commune health station. On average, each health staff is responsible for managing and implementing 2 to 3 targeted programs at the commune health station, while also sharing the responsibilities of occasional single-day events, such as periodic mass immunizations and/or infectious disease outbreak investigations and containment as necessary. While there has been a study regarding primary health care system capacity response to NCDs in rural areas of Vietnam [13], a similar study in urban settings has not yet been conducted or published. In response to increasing NCDs, a pattern of growth compounded by the challenges of rapid urbanization on the health care system, it is necessary to explore the status of primary health care services at commune health stations. Thus, this study aims: 1) to explore the views of health professionals on the responsiveness of primary health care services in addressing NCDs at the commune health stations within an urban setting, and 2) to identify areas of improvement for urban NCD primary health care service delivery.

## Methods

### Study setting

This study was conducted in Hanoi, the capital city of Vietnam, which comprises of 30 districts, including 12 urban districts, one district-level town (Son Tay) and 17 rural districts. Each district is divided into wards and towns, which are equivalent to communes. The population in Hanoi was estimated to be 6.9 million in 2015, of which 2.9 million (42%) lived in urban districts [14]. The typical urban districts are located in the four central districts of Hanoi. They are densely populated and include both slum and non-slum areas. These urban districts have 73 commune health stations, of which 32 commune health stations are involved in the national program on NCDs (16 commune health stations with the hypertension program and 16 commune health stations with diabetes program). In this study, we focused on four commune health stations, located in two urban districts within Hanoi’s city center. These commune health stations are responsible for providing primary health care for the population in their communes whom are representative of the urban population of interest for this study.

### Analytical frame and scope of study

**Fig. 1:**
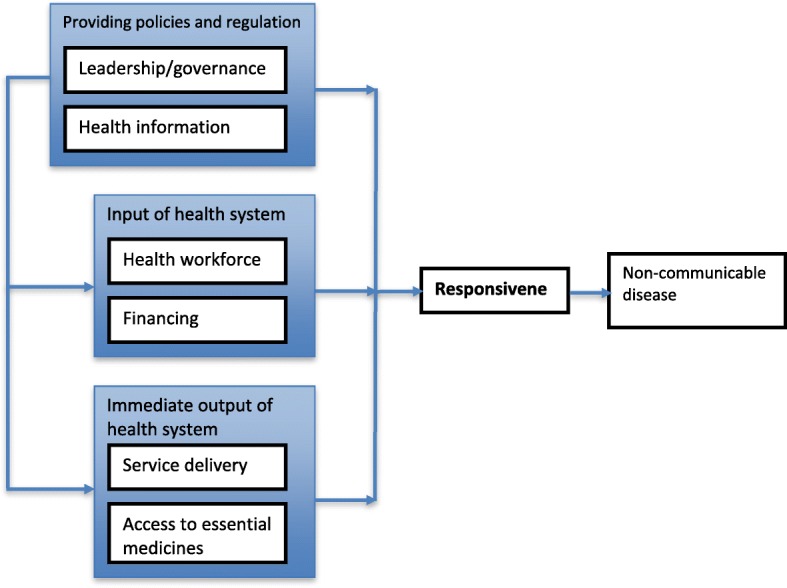
The adapted analytical framework for the responsiveness of commune health stations to non-communicable disease

The health system framework proposed by the WHO includes six building blocks used in the monitoring of health systems, including 1) service delivery, 2) health workforce, 3) health information systems, 4) access to essential medicines, 5) financing, 6) leadership/governance [15]. It is notable that the health system components framework has areas of overlap: leadership/governance and the availability of a health information system provides the basis for the overall policy and regulation of all the other building blocks, while financing and health workforce are key input variables to the health system. Further, access to essential medicines and service delivery reflect the immediate outputs of the health system. In this study, we adapted the WHO health system framework to explore how health professionals view the responsiveness of commune health stations towards NCDs in an urban setting. Figure 1 presents the analytical framework, with a simplification of the six building blocks of the health system framework to address responsiveness and to connect primary health care responsiveness to NCDs. The scope of the study was limited to the perspective of health professionals on primary health care services for NCDs at the commune health stations. In addition, we only focused on NCDs, including cardiovascular disease, diabetes, cancer and chronic respiratory diseases.

### Study design and sampling

We undertook a qualitative approach using thematic analysis [16] to synthesize the views of health professionals on primary health care services to NCDs. Qualitative data was collected through in-depth interviews. In-depth interviews are considered appropriate tools for collecting rich information that can provide evidence to policy makers [17]. Thus, it was considered a suitable tool to study the opinions of health professionals, the results of which can be used to guide local policy changes. By asking open-ended questions, we were able to explore in-depth the ideas and information provided by each participant.

The study was conducted between July and August 2015. A total of 19 in-depth interviews were conducted. We purposively selected two districts in order to cover different areas in urban Hanoi. One district represents regions within the old quarter, and the other represents both old quarter and new urban areas. In addition, these two urban districts consist of typical slum areas, which are in close proximity to non-slum areas [18]. A comprehensive list of all commune health stations in these two districts was collected from their district health centers. In these two districts, there were two types of commune health stations: stations involved in the national program for NCDs (either hypertension or diabetes) and commune health stations without any targeted NCD programs. Although the health system in Vietnam was decentralized, and the targeted program on NCDs at the commune level was at a pilot stage, we sought to explore the variability between a commune with and without the national targeted program on NCDs. In each district, two commune health stations were selected, including one commune health station with an NCD program and one without any NCD program. In each selected commune health station, we purposively selected participants involved in NCD specific health-related activities, including physicians, physician assistants, pharmacist assistants and nurses. To supplement information from the commune level, we also interviewed NCDs managers at national, provincial, and district levels regarding the implementation of primary health care services for NCDs at the commune level.

### Study tools and data collection

Two interview guides were developed: one for NCD managers at different levels and one for health staff at commune health stations (Additional file 1). The interview guides were written in Vietnamese. These guides were nearly identical, except for the inclusion of one additional question related to the availability of health information systems technology for NCD at the participant’s level specifically for NCD managers. The interview questions were guided by the WHO conceptual framework for health systems, adapted for relevance to the current situation in Vietnam’s primary health care context. The interview questions were classified into two sections: the first section focused on personal and professional information such as the participants’ age, sex, work position, work experience and educational background; and the second section was designed to explore participant views on primary health care services based on the WHO’s six building blocks. In addition, the interview questions included a question about the vital needs of commune health stations to improve their primary health care service for NCDs. Our interviews were carried out in private rooms at the commune health station. For interviews with participants at the district, provincial and national levels, our interviews were carried out at their offices. All our interviews lasted between 45 min and 1 h. The first author conducted all interviews with the support of one research assistant. Since no relevant new information appeared concerning our research questions, saturation of information was judged to have been reached after these 19 interviews.

To complement data from the interviews, we conducted a brief inventory of NCD related activities at the four selected commune health stations. A checklist of existing NCD-related activities was developed and used to collect information from relevant health staff at the commune health stations. This checklist contained questions about the number of NCD patients per month, the availability of equipment and medicine for NCD treatment, the availability of preventive and curative NCD activities, and the skills of staff for NCD prevention and treatment.

### Data management and analysis

The interviews were audiotaped and transcribed verbatim into MS Word by a research assistant. The transcripts were reviewed and corrected by the first author. All transcripts were then translated into English by the first author and shared among co-authors for review. The data were entered into the OpenCode version 4.02 software [19]. An initial descriptive coding framework was developed based on the WHO framework for health systems [15], implying that data was organized to these pre-determined themes, i.e. themes were initially identified in a theoretical, deductive way [16] . We manually reviewed the verbatim interview transcripts multiple times, and developed codes to capture the content of the responses from each participant [16]. Subsequently, the codes were grouped into sub-themes and organized under our core themes, i.e. the WHO framework for the six blocks of the health system (Table 1). Beyond these six pre-determined themes, an additional theme describing areas of improvement for primary health care NCD services was inductively identified based on our data. Thus, we combined our theoretical thematic analysis with an inductive approach [16], to remain open to the emergence of additional relevant themes from our data. We finalized the overall themes by developing subheadings that captured and summarized the content and main ideas within each of the themes. The final thematic categories were refined following discussion with other members of the research team [16]. The results of the inventory were summarized in a table and compared against the themes identified for the interviews.

**Table 1:** One example of the data analysis process using thematic analysis approach

Code	Sub-themes	Themes
Limited understanding about NCD policies and strategies	Unawareness of the NCD strategies	Leadership/governance-Unawareness and weak implementation of national strategies
Don’t know about any NCD policies	Unawareness of the NCD strategies	
Know some policies about NCDs, but too busy to focus on this issue	Weak implementation	
No guideline for NCDs at commune health stations	Weak implementation	

## Results

### Social demographic characteristics

A total of 19 participants took part in the study. Table 2 shows the distribution of age, gender, professional role, work experience, job title and qualification of participants in the study. The age ranged between 25 and 54 years, of whom half were between 25 and 34 years of age. Most participants were female (17/19), which reflected the current predominance of female health professionals within Vietnamese commune health stations. Their work experience ranged from newly graduated to those almost nearing their retirement; most participants had worked more than 5 years (17/19).

**Table 2:** Number and distribution of participants by age group, sex, job title, work experience and qualification

Characteristic	Frequency
Age group (years)	
25–29	5
30–34	5
35–39	2
40–44	2
45–49	3
50–54	2
Total	19
Sex	
Male	2
Female	17
Total	19
Work experience (years)	
< 5	2
5–9	7
10–14	3
15–19	3
> 20	4
Total	19
Professional role	
Medical doctor	8
Doctor assistant	4
Pharmacist/pharmacist assistant	3
Nurse	4
Total	19

The analysis resulted in six themes describing the responsiveness of commune health stations to NCDs and one theme describing needs for improvement for primary health care NCD services. The first six themes were deductively developed based on the WHO framework while the seventh theme was inductively developed based on our data. Below we describe these themes in detail and exemplify with quotes from the interviews how these themes were reflected in our data.

### Service delivery – Unsystematic, limited and inadequate

The primary health care services for NCD were acknowledged to be on a limited scale, with unsystematic implementation.

*“Because services related to the management of NCDs have not been implemented systematically at primary health care levels, the coverage has been limited, so the proportion of people at high risk of accessing health services were small, and this doesn’t ensure health equity.”* – (health staff at the national level).

Inadequate quality and quantity of primary health care services for NCDs at commune health stations were noted by NCD managers at the district level. The national target programs on NCDs were implemented in only a few communes within a district, and only offer health care provisions to a small number of the population.

*“… [in our district] we implemented the hypertension program for only 4 communes and implemented the diabetes program for 4 other communes [among 18 communes]. We don’t have any NCD programs for the rest of communes...”* – (health staff at the district level).

Most health staff mentioned that they knew the burden of NCDs among their population. However, they lacked autonomy to implement NCD programs, or to provide more primary health care services at their commune health stations.

*“...some common NCDs increased in our commune, but we could not provide any primary health care service here. The problem is we lack human resources, medicines and equipment.”–* (health staff at a commune health station without any national target program on NCDs).

However, some health staff at the commune health stations also strongly argued that their workday was already overwhelmed with professional responsibilities, such that they did not want to provide NCD services at their commune health stations. Likely, there was a concern regarding declining quality of services if the scope of the services was expanded.

*“As I told you, we cannot implement health care service for NCDs. We have many tasks, and we lack a doctor here.”*- (health staff at a commune health station without any national targeted program on NCDs).

### Leadership/governance- lack of awareness of and weak implementation of the national strategy

NCD managers at the national, provincial and district levels highlighted a national strategy for NCDs issued by the Prime Minister targeting cancer, CVDs, diabetes, COPDs, asthma and other NCDs for the period between 2015 and 2025. This strategy legally obliged the country and relevant stakeholders to address the problem of NCDs. In addition, the strategy also focused on risk factors for NCDs, including smoking, alcohol abuse, food safety and low levels of physical activity. However, even if the national strategy was acknowledged at higher levels, health staff at commune level were not aware of its existence, nor its practical applications. All participants at the commune health stations lacked awareness regarding the national strategy for NCDs.

*“I don’t know. It’s too macro level when talking about national strategy or policies”-* (health staff at a commune health station with the national program on NCDs).

Even at commune health stations involved in the national program for hypertension or diabetes, health staff did not know about the national strategies for NCDs. Health staff expected NCD managers at their commune health stations to know; however, NCD managers were not aware either.

### Health information- limited, fragmented and inadequate for planning

Health information is considered crucial for health planning at the national level. However, it was noted that the information was limited, fragmented and inadequate, since national target programs collected data and even so, only sporadically and on a small scale. The national target programs on NCDs cover only a few NCDs, such as hypertension, diabetes, COPD and cancer.

*“The health information system is very important, however, NCD reports were done only through the national target programs, and there wasn’t a unique system yet. It means that NCD-related statistics and data are limited, fragmented and inadequate” –* (health staff at the national level).

The lack of data on NCDs was also noted at the district level. Since NCD patients frequently go to hospitals, and hospital systems lack a comprehensive centralized data-sharing system, there is insufficient coordination of information between the curative and preventative health systems in Vietnam. Usually, commune health stations report on NCDs based on the NCD target programs; however, as the commune health station staff had to manage multiple priorities, the quality of the reports typically suffer from divided attention.

*“We don’t have much information because we are not a curative facility. We had information about NCD from four* commune health stations *participating in the national program, and our two clinics for health insurers. For other* commune health stations*, we don’t have data about NCDs”* – (health staff at district level).

Most health staff at commune health stations mentioned the lack of NCD data, however, they all referred to and expected NCD managers to answer specific questions about NCD data. It is the NCD manager at a commune health station that is assigned the duty of collecting NCDs information and preparing relevant reports. NCD data were reported within the national target program, such as hypertension and diabetes.

*“The quality of NCD data was not high because we did not collect all NCD cases. We only collect data within our program*”- (health staff at a commune health station with the hypertension program).

The NCD data were from a limited number of patients enrolled in the treatment programs. Consequently, epidemiological data is limited by the absence of a system to collect disease burden information from those not enrolled in treatment at a commune health station. The low quality of NCD reports was again confirmed at the commune level. The NCD data were collected passively based on self-reported NCDs from patients, or those of health collaborators.

*“For me, the NCD reports were collected very passively. For example, if patients present to the clinic, then the diagnosis occurs. That is how and when we record their information and diagnosis in our ledgers”-* (health staff at a commune health station without any national program for NCDs)*.*

*“We have monthly and quarterly reports for NCDs. But we just recorded patients who visited the clinic. In addition, we got information from our health collaborators when they knew someone in their community was diagnosed with a NCD. So, our data about NCDs are underestimated, and incorrect”-* (health staff at a commune health station).

### Health workforce –misallocated and insufficient capacity

Insufficient workforce targeting NCDs at commune health stations in terms of number of staff and technical training was widely mentioned by participants. The national target programs on NCDs have not been extended to include all commune health stations, so in general, there are discrepancies between funding and technical support for primary health care services provisions for NCDs at commune health stations generally.

*“For the health workforce at* commune health stations*, some facilities either lack human resources and/or lack capacity. They need to be strengthened in their capacity to provide services for NCD prevention, consultation, early detection, and management. The reason is we haven’t implemented NCD services systematically at the primary health care facilities.”-* (health staff at the national level).

Misallocation of health staff at commune health stations was mentioned. While there are a lot of nurses, midwives, and pharmacist assistants, medical doctors are lacking. Particularly at commune health stations, there was an insufficient number of medical doctors dealing with NCDs. NCD managers at a district level strongly emphasized the limited incentives to the recruitment of medical doctors to commune health stations, due to low salaries as well as fewer professional development and career advancement opportunities.

*“… [at* commune health stations*] there are a lot of nurses, midwives and pharmacist assistants, but we lack medical doctors. Generally, there is one medical doctor per* commune health station*, but some* commune health stations *do not have medical doctors. In addition, a medical doctor at the* commune health station *normally holds leadership positions at the* commune health stations and therefore, has *many responsibilities beyond clinical work. Actually, what we need are staff responsible for clinical duties, who are trained as a medical doctor or a physician assistant.” –* (health staff at the provincial level).

*“We are lacking professionals at* commune health stations*. However, it is hard to recruit a medical doctor to work for* commune health stations*, especially those who have specialized skills in NCDs” –* (health staff at the district level).

Most health staff at commune health stations also confirmed that their commune health stations had enough health staff but suggested that their capacity on NCDs was not sufficient to meet the requirements of the population. The ancillary staff also discussed a need for more specialized training on NCDs for support staff.

*“… I think that our capacity [on NCDs] would not meet the requirement of patients because patients do not want to visit us. Their demand is higher that our capacity”-* (health staff at a commune health station without any national program on NCDs).

At commune health stations without any nationally targeted programs on NCDs, most participants noted that they had enough staff because they did not provide any primary health care services on NCDs.

*“We have enough staff. But, it’s hard to say about capacity. So far, I’ve found that we worked well because we did not have any NCD services”-* (health staff at a commune health station without any national programs on NCDs).

### Financing – Limited budget for NCD services

Insufficient public financing for primary health care services of NCDs was emphasized by most participants. The budget for NCDs was available only for communes with national target programs; there is no mechanism to use health insurance at a primary health care level for NCDs. In addition, the national target programs on NCDs are anticipated to undergo a reduction in the near future, which would further limit the funds available for NCDs.

*“The budget for primary health care services of NCDs is very limited; [funding is] mainly through the national target programs on NCDs, but the programs have been cut down. There are some barriers within health insurance reimbursement for NCDs at primary health care level”-* (health staff at the national level).

The lack of a budget for NCDs was also noted by NCD managers at the district level. This led to a shortage of medicines as well as reduced coverage of primary health care for NCDs at commune health stations.

*“The budget was so limited for our commune health station…. In our 4 communes with NCD programs, in the first round, many patients participated, but [participation] reduced year by year. That’s because we lacked medications, and there was a poor response to treatment, so [patients] quit the programs.”-* (health staff at district level).

*“We always want to get more funding. Historically, the funding for NCD has always been limited. And…, there weren’t enough medications. So even if patients request treatment, we cannot provide the medications”-* (health staff at district level)*.*

However, some health staff at commune health stations interpret the absence of medications as secondary to a broader systemic issue. These participants reported that because district health centers are responsible for dispensing medications, NCD budgets do not affect medication availability at a commune health station. In addition, commune health station staff expressed a desire to maintain autonomy in budgeting at commune health stations, to reflect the local health needs.

*“It’s a bit difficult to say. I am a staff member, so I don’t know. All of the large decisions are made by higher levels.” –* (health staff at a commune health station without any national program for NCDs)*.*

*“I know that medications [for hypertension program] are provided by the district health center. So, I don’t see any impact of providing NCD services on the budget [at the commune health center level]” –* (health staff at a commune health station with national program on NCDs).

Some health staff at the interviewed commune health stations requested more funding for NCDs. In general, staff expressed that low salaries were also an obvious limitation for an overwhelmed health system.

*“We always want to have more funding [for NCDs], but we don’t know how to get more.” –* (health staff at a commune health station without any national program on NCDs)*.*

*“For me, it’s necessary to provide additional funding for health staff that improves staff satisfaction… I’ve found the incentive is not commensurate to our labor. We love our work here, but the salary was not sufficient.”-* (health staff at a commune health station with the national program on NCDs).

### Access to essential medications – Either absent or insufficient

NCD managers at the national level noted that the essential medications at commune health stations include provisions for some NCD medicines.

*“The essential medications, including hypertension and diabetes medications, were in the list of available medications for* commune health stations *[by regulation].* Commune health stations *have sphygmomanometer [for measurement of blood pressure], but they don’t have equipment for the rapid test of blood glucose. Medications for COPD and cancer are not available at* commune health stations*.”* – (health staff at the national level).

However, health staff at commune health stations reported that they did not have any medications for NCDs at their commune health stations, especially in commune health stations without the national target program for NCDs. For commune health stations within the national target programs on hypertension, the district fund and provide medications, which are distributed to enrolled patients via the commune health stations. For commune health stations within national target programs on diabetes, only screening is currently provided, as treatment is not yet available. These highlight the spectrum of NCD management regarding specific diseases and their variable availability within communes.

*“…We do not have medications at our* commune health stations*. What we have are some medications specifically for emergencies”-* (health staff at commune health stations without any national target program on NCDs).

*“…We receive medications for hypertension from the district level for patients enrolled in the program [national target program on hypertension] in our commune. There is no medication for other NCDs because we do not provide any NCD services here”-* (health staff at commune health stations with the national targeted program on NCDs).

At commune health stations within the national target program on hypertension, shortage of medications for hypertension was noted by NCD managers at provincial, district and commune level. Delay in the bidding process was the main reason for the shortage of medications. In these situations, patients must privately purchase medicine using out-of-pocket funds or forego medication usage entirely.

*“Normally, the shortage happens about 3 months in a year. Our doctors still prescribe for our patients and ask them to buy [the medications] themselves using their own funding”* – (health staff at the provincial level).

*“Sometimes we lack hypertension medications because the district did not provide medications for us*.”- (health staff at a commune health station with the national target program on NCDs).

### Needs for improving primary health care services for NCDs – Budget, basic data collection and professional training for implementing NCDs services

Table 3 shows the summary of recommendations to improve primary health care services for NCDs at commune health stations. Most participants requested additional funding support.

**Table 3:** Summarizing vital needs to improve primary health care for NCDs at commune health stations

*Recommendations for improving NCD services at* commune health stations 1. Provide more budget to implement NCD related-services 2. Recruit more medical doctors, especially those who have specialization on NCDs 3. Invest more equipment to aid in diagnose and early detection of NCDs 4. Provide more professional training for health staff, e.g. diagnosis, treatment, and communication with NCD patients. 5. Make medicines for NCDs available so commune health stations can provide treatment directly 6. Implement health insurance support for NCDs at commune health stations 7. Develop a service package for NCDs at the commune level for eligible for coverage by the national insurance program	

*“We need additional funding to provide incentives for our health collaborators. In addition, funding can help us provide [more] information about NCDs to the community through workshops and other forums. Finally, we need increased funding to directly increase our salary because it’s difficult to perform well with a low salary”-* (health staff of a commune health station at a commune health station with the national program on NCDs).

*“We need more funding to implement surveys to understand the status of NCD in our community. We also need funding to provide an incentive for our health collaborators, including people from organizations who help with implementation and execution of field activities. Besides that, we need training materials for staff on NCDs and equipment for NCDs management” –* (health staff at a commune health station without the national program on NCDs).

Most participants also noted the importance of more human resources, training, and equipment at the commune health stations. Some participants stated that increasing medication availability for NCDs at the commune health stations was important to help commune health stations actively provide primary health services for NCDs. Notably, some participants recommended that health insurance be implemented at commune health stations with the development of a service package for NCDs at commune health stations.

### Brief inventory of NCD activities at four selected commune health station

Staff at the four selected commune health stations were also asked to fill in a checklist of the scope of each commune health station’s involvement in NCD management. These are documented in Table 4. Overall, few patients visited the commune health stations every month. Medications and equipment for NCDs were lacking. Limited preventive and curative NCD interventions were available at the commune health stations. All commune health stations reported inadequate training for NCD prevention and treatment.

**Table 4:** Inventory non-communicable disease activities at four selected commune health station

	Hypertension	Diabetes	COPD	Cancer
Number of patients per month/commune health station	5–10	2–3	0	0
Number of commune health station with relevant equipment	4/4	1/4	0/4	0/4
Number of commune health station with relevant medicine	0/4	0/4	0/4	0/4
Number of commune health station with preventive NCD activities	2/4	2/4	0/4	0/4
Number of commune health station curative NCD activities	1/4	0/4	0/4	0/4
Number of commune health stations with adequate skill for NCD prevention and treatment	2/4	2/4	0/4	0/4

*NCD* Non-communicable disease

## Discussion

This study explored the ability of commune health stations to respond to NCDs through the perspective and experiences of health professionals in urban Hanoi. Their verbatim accounts provide insight on the various health system factors which may complicate the delivery of NCD services at commune health stations. These issues were explored through application of the six WHO building blocks of the health system, including governance, health information, health workforce, financing, service delivery and medications [15]. The findings from our study showed that commune health stations had not been prepared to respond to the rising prevalence of NCDs in urban Hanoi, Vietnam.

The Vietnamese government recognized the burden of NCDs early on, in 2002 [20]. The country has developed and implemented several policies and strategies for prevention and control of NCDs and their risk factors [12, 20]. These policies and strategies were implemented via the establishment of national target programs on NCDs. Although the national target programs on NCDs have been conducted nationally, their coverage has been limited and is still lacking prioritization by local authorities [12]. Our findings showed that NCD managers at higher levels were aware of the national strategy on NCDs. However, most participants at commune health stations, those responsible for enacting the day-to-day patient education and management, were unaware of the national strategy on NCDs. This demonstrated a lack of policy dissemination from higher levels to the grassroots level. In addition, since commune health stations did not implement NCD services, health staff at commune health stations are not up-to-date in their knowledge and practices regarding NCDs. Although relevant policies were available on a national level to address the problems of NCDs, it is essential to plan and implement cost-effective intervention via local health facilities [21].

We found that higher level administrative health staff recognized the inadequacy of health information technology for planning and implementing interventions. Among participants at commune health stations, NCD data collection is described as passive, and did not accurately reflect the incidence of disease within the local community. This finding is consistent with the results from the joint annual review regarding NCDs in Vietnam suggesting that the quality and timeliness of NCD reports were not sufficient for management and planning [12]. The lack of NCD information is likely to impact the development of evidence-based NCD policies and interventions. In addition, an effective health information system would strengthen the population’s.

health, improve accurate resource distribution, and enhance management capacity [9].

Workforce insufficiency impacts the availability of primary health care services for NCDs at commune health stations. To provide effective NCDs services, the health care workforce needs to have appropriate education and training [21]. As noted by most participants, since there is an insufficient health care workforce, along with the absence of skilled and specialized providers, it has been impossible to provide primary health care services at commune health stations. In addition, given the multitude of health programs implemented at a commune health station at any given time, a well-trained staff would not have sufficient time to focus on NCD service provision, particularly as it requires a preventative focus. In a study from a rural district of Vietnam, Minh et al. also found that the quality and quantity of health staff were insufficient at primary health care level [13]. Evidence showed that the human resources for NCDs were planned specifically to meet NCDs needs, and consequently, there was an effect on NCD strategy monitoring and implementation [13, 22, 23].

Together with an insufficient, under-trained health care workforce, health financing is a key component to an improved health care delivery system [15], which in turn impacts the implementation of NCDs interventions. As noted by participants, the lack of a budget prohibited staff from conducting NCDs interventions commensurate to the burden of disease within the community. While a state budget for NCDs prevention, screening, and diagnosis were allocated to some commune health stations participating in the national target program on NCDs (e.g. hypertension or diabetes), the budget has been very limited. In addition, since commune health stations were not included in the health insurance scheme [12], they did not have any other allocated budget to implement NCDs interventions for patients within in their communes.

We found that almost no primary health care services for NCDs were conducted at the commune health stations. Since there were only a few communes involved in the targeted national programs on hypertension or diabetes (i.e. 4–5 in each district) one could infer that this number reflects the overall situation in urban Hanoi. Further, even at those two commune health stations involved in the targeted national programs, screening services and treatment were implemented for a limited population only. This is in direct contrast to recommendations that primary health care services for NCDs be implemented at the primary health level [9]. Since patients with NCDs require longitudinal care, primary care can deliver better health outcomes at a lower cost [7]. As the prevalence of NCDs is concentrated among the poor in both slum and non-slum urban settings [18], and urban populations also have a lower utilization of commune health stations [24], socioeconomic inequalities may increase if the country does not address the burden of NCDs in an appropriate and timely manner. Thus, it is necessary to strengthen the primary health care services for NCDs so that patients with NCDs can be managed more locally by urban commune health stations instead of tertiary hospitals. Moreover, to maximize the commune health stations participating in NCDs programs, there should be better selection criteria to account for comorbid diseases, such as diabetes and hypertension. To successfully address the burden of NCDs, the integration of NCDs program into other health programs at a primary level should be more robust [7, 23, 25, 26].

Ensuring patients with NCDs access to essential medications at the primary health level is a necessary foundation to control and manage NCDs successfully [9]. Although Vietnam has an essential drug list and a health insurance drug list including most medications needed for NCD treatment [12], this study found that some commune health stations had no NCD treatment medications available. In some commune health stations participating in the national target programs on NCDs, NCD treatment drugs were provided by the district level to a limited number of registered patients via the commune health stations. In addition, the commune health stations in our study did not accept the national health insurance, so patients with NCDs in these regions were not eligible to use their health insurance cards at these commune health stations. Evidence showed that the responsiveness of commune health stations to NCDs would be impacted by the absence of NCD treatment drugs [22, 23, 27]. The lack of NCD treatment drugs prevented commune health stations from providing primary health care services for NCDs [9].

Overall, the findings of this study complement the results in reports by the Ministry of Health, which showed that health information, human resources, health financing, service access and medications for NCDs were insufficient in Vietnam [12]. Our findings were also consistent with a study in urban Vietnam highlighting the inadequacy of the primary health care system to serve the NCD-related health needs of the population [13]. In the South East Asia region, Bart et al. also found that the primary health system in Cambodia was unable to manage NCDs, even though the burden of NCDs was increasing [28]. Having reviewed several papers on the health system and NCDs, Priya et al. reported similar results that there is a gap in health systems regarding NCDs in Asian-Pacific territories [29]. In Vietnam, although there are national programs for NCDs, these programs are limited in scope and targeted population. In addition, the current health insurance plan, which is meant to be a safety network for patients without coverage, did not cover NCD coverage at the commune health station (these must be accessed at the district hospital level). The lack of a health insurance package for NCDs has prevented commune health centers from providing the NCDs services, including screening, early diagnosis, treatment, and management, to meet the needs of the local community.

### Trustworthiness of the study

Trustworthiness in qualitative research is mainly judged by the ability of the study to capture what it really intended to explore [30]. This requires careful consideration throughout the research process from its design to the final results. In this study, trustworthiness was ensured by the first author’s deep involvement throughout the research process, which enabled the researcher to come “close to the study subjects” [30]. The first author, who has a background as a medical doctor, facilitated the data collection process as he was deeply familiar with the role of health staff in each level of the medical system. Because the discussion focused only on the participants’ work, biases related to personal issues likely did not impact on the interviews. In addition, the information provided by commune health stations was cross-checked through interviews with participants at the district, provincial and national levels. All interviews were planned, conducted and analyzed by the first author. In addition, credibility was ensured by triangulation amongst investigators, with analysis conducted through collaboration between the first and the last authors along with additional input from the rest of the research team. This allowed perspectives from several different angles to be brought up and discussed, with both “insider” and “outsider” perspectives on the Vietnamese health system. In addition, interview data was complemented and mirrored against a standardized inventory of NCD related activities at the four selected commune health stations. Further, we have tried to describe each step in the research process in detail, for others to be able to follow and judge the soundness of our results.

This study provided an opportunity to rigorously understand how health professionals view the responsiveness of primary health care services for NCDs at commune health stations. However, the findings must be interpreted with caution. In this study, we included only four commune health stations and 19 participants (including 4 participants from higher levels). Although the findings provide valuable insights into the responsiveness of commune health stations to NCDs in Hanoi, the findings are not evidently generalizable to other urban settings.

However, by describing our study context in detail, we think that others will be able to judge whether these results are also transferable to other similar contexts. We purposively selected districts and health centers that are “typical” and reflective of urban Hanoi to gain in-depth information about how health professionals view the responsiveness of commune health stations to NCDs. In qualitative research, the sample is often small and demographically non-representative [30] . The advantage of using qualitative interviews is that we could explore their views in depth and from their own perspectives by using open and flexible interview guides, rather than pre-determined questions and answers. Thus, instead of aiming for statistical generalization or representation, our qualitative research aimed to achieve analytical generalizations, i.e. derive results with “theoretical” application to other contexts. Further, since planning in Vietnam follows a top-down approach, commune health stations normally follow the guidelines and direction from district and provincial levels. This implies that commune health stations implement hypertension and diabetes programs in the same manner in all pilot commune health stations. Further, we complemented interview data with an inventory of existing NCD activities at these health centers, which confirmed data gained from the interviews. In addition to the staff at the selected commune health stations, we also interviewed managers at the district, provincial and national levels, which also provided perspectives beyond the four selected health stations and offered a more generalized view of the medical system. This supports the partial applicability of these results to other urban settings in Hanoi.

## Conclusion

There are different perceptions among NCD managers at higher levels and health staff at commune health stations in terms of availability of the national strategy for NCDs. However, most participants agreed that the NCDs programming at commune health stations in urban Hanoi are weak, with limited health information, sparse human resources, poor financing, inadequate quality and quantity of services, and lack of essential medications. Our recommendations for improving the primary health care services for NCDs at commune health stations include: providing more funding to implement NCD related services, including collection of basic health information/data; NCD workforce development to increase human resources; and providing equipment and medicines for NCDs. A comprehensive service package for NCDs should be developed so that access to primary health care at commune health stations can be covered by the national health insurance.

## Additional file

Additional file 1: In-depth interview guide: health staff. (DOCX 16 kb)

## Abbreviations

COPDs: Chronic obstructive pulmonary diseases
CVDs: Cardiovascular diseases
GDP: Gross domestic product
HIV: Human immunodeficiency virus
LMICs: Low- and middle- income countries
NCD: Non-communicable disease
WHO: World Health Organization
