# Supplementary material for: Views by health professionals on the responsiveness of commune health stations regarding non-communicable diseases in urban Hanoi, Vietnam: a qualitative study
Source: BMC Health Serv Res. 2018 May 31;18:392. doi: 10.1186/s12913-018-3217-4 (PMC5984436; doi:10.1186/s12913-018-3217-4)
Supplement: Supplementary file 1 — In-depth interview guide: health staff. (DOCX 16 kb) [file 12913_2018_3217_MOESM1_ESM.docx]

# Annex 1: In-depth interview guide: health staff

## General information of interviewee

Full name: Sex: Male/Female Age:

Year of graduation: Number of years in his/her work:

Position: doctor/doctor assistant/pharmacist/pharmacist assistant/lab technician/nurse/NCD manager

Level: National/Provincial/District/Commune

1. Could you please introduce yourself and your role/profession?
   - When did you graduate? How long have you been working here?
   - Please tell me about your background etc.
2. How would you describe an ordinary working day – what do you do (ask for details and examples)?
   - How do you think your training meets your needs at work?
3. How do you define NCDs? Please list NCDs you know. Which of these NCDs would you label as the biggest problem in your country/province/district/commune? Please explain why.
4. In regards to a patient with (NCD) that you mentioned – what health services/primary health services do you think should be available at commune health center? And what NCD services are actually implemented at the commune health centers in your region? Please describe.
5. To what extent have each NCD services been implemented in your region? What, if any, guidelines are available for these issues?
   - What worked well to meet the needs of your target population? Please elaborate.
   - How do you see the accessibility of these NCD services (equally)? Please explain.
   - What are your thoughts about the extent of their coverage? and efficiency? please explain.
   - What could be improved? Please explain why.
6. What strategies and policies related to primary healthcare services for NCDs are you aware of? How do they relate to your current work on NCD patients? What strategies and policies should be added to improve the primary health services for NCDs? Please explain why.
7. To what extent do you see the budget impacting primary health services for NCDs? How do NCD patients pay for health services at commune health centers? What barriers exist to out-of-pocket healthcare payment? Please elaborate.
8. How do you describe the health workforce for NCDs at commune health centers in term of size and capacity? What burden exists to NCD disease specific care? Please explain.
9. What medicines and drugs/technology are available for NCD diagnosis/treatment and prevention at commune health centers?
   - How do they meet the needs of target population? Please explain.
   - How do you perceive the quality of them?
   - What is their accessibility? What limitations exist based on income or insurance coverage?
10. Please describe the health information system for NCDs in your region.
    - What needs exist and how does this health information system meet those needs? Please explain.
    - How do you perceive the quality of health information data?

*[This question was used if the interviewee was a NCD manager]*

1. What are the most vital needs and arrangements of commune health centers that if provided would ensure an increase in the capacity of NCD management in your region?
2. Is there anything you would like to add?
